# Trace DNA Transfer in Co-Working Spaces: The Importance of Background DNA Analysis

**DOI:** 10.3390/ijms25042207

**Published:** 2024-02-12

**Authors:** Martina Onofri, Federica Tommolini, Simona Severini, Cristiana Gambelunghe, Massimo Lancia, Luigi Carlini, Eugenia Carnevali

**Affiliations:** 1 Forensic Sciences Laboratory, Section of Legal Medicine, Department of Medicine and Surgery, Santa Maria Hospital, University of Perugia, 05100 Terni, Italy; f.tommolini@aospterni.it (F.T.); s.severini@aospterni.it (S.S.); e.carnevali@aospterni.it (E.C.); 2 Section of Legal Medicine, Department of Medicine and Surgery, University of Perugia, 06123 Perugia, Italy; cristiana.gambelunghe@unipg.it (C.G.); massimo.lancia@unipg.it (M.L.); luigi.carlini@unipg.it (L.C.)

**Keywords:** forensic, touch DNA, non-self DNA, background DNA, DNA transfer, prevalence, office, co-worker, partner

## Abstract

The presence of background DNA (bgDNA) can hinder the evaluation of DNA evidence at the activity level, especially when the suspect is expected to be retrieved due to their habitual occupation of the investigated environment. Based on real-life casework circumstances, this study investigates the prevalence, composition, origin, and probable transfer routes of bgDNA found on personal items in situations where their owner and person of interest (POI) share the same workspace. Baseline values of bgDNA were evaluated on the participants’ personal items. Secondary and higher degree transfer scenarios of non-self DNA deposition were also investigated. The DNA from co-workers and co-inhabiting partners can be recovered from an individual’s personal belongings. Non-self DNA present on the hands and deposited on a sterile surface can generate uninformative profiles. The accumulation of foreign DNA on surfaces over time appears to be crucial for the recovery of comparable profiles, resulting in detectable further transfer onto other surfaces. For a thorough evaluation of touch DNA traces at the activity level, it is necessary to collect information not only about DNA transfer probabilities but also about the presence of the POI as part of the ‘baseline’ bgDNA of the substrates involved.

## 1. Introduction

The proven attribution power of forensic genetics, coupled with the enhanced sensitivity of profiling techniques, allows the detection of small amounts of biological evidence (trace DNA) and the identification of its contributors [1,2]. Especially with regards to trace evidence, genetic material from an individual found at a crime scene does not necessarily prove their involvement in the alleged criminal activity. This could be due to deposition predating the event, indirect transfer during the disputed act, or a legitimate activity. Therefore, since retrieved genetic evidence may or may not be pertinent to a criminal proceeding, the criminal justice system is progressively seeking the assistance of forensic experts to address questions regarding the likely timing and circumstances of a trace’s deposition [3,4,5,6]. The statistical assessment of the strength of DNA evidence given alleged activities that could have resulted in the trace’s deposition is referred to as activity-level reporting (ALR) [3,5,7]. ALR is performed based on case-relevant variables resulting in often-times complex probabilistic inferences, which can be greatly facilitated by the use of Bayesian Networks (BNs) [8,9]. BNs are graphical representations of said variables, their dependencies, and respective probabilities. Thus, taking into account case-specific factors and variables, the forensic expert must gather probability values of DNA transfer, prevalence, persistence, and recovery (TPPR) either from peer-reviewed and published data reflecting case-relevant elements, or from in-house experiments, ideally replicating realistic casework conditions [5,6,7,10,11,12,13,14]. Data produced under strictly controlled laboratory conditions, while informative for the specific variable investigated, may lack the complexity observed in casework evidence. One such example is the possible presence of numerous contributors to the trace, which could be the result of multiple persons of interest (POIs), the detection of background DNA, or both. The term background DNA (bgDNA) refers to DNA of a known or unknown origin, not attributable to the individuals that, given the case circumstances, are expected to be present as contributors, such as the POI [5,11,15,16,17]. bgDNA, also referred to as non-prevalent DNA, since it is not relevant to the disputed event, can be deposited before, during, or after the activity in question, and may originate from various sources and different mechanisms. Individuals constantly release their DNA into their surrounding environment, through shedding skin cells, depositing saliva droplets, or by touching their surroundings, whilst simultaneously collecting on their person foreign, non-self DNA [18,19,20,21,22,23,24,25]. The deposition of DNA from various individuals to their surroundings has been observed in places they habitually or occasionally occupy, as well as in spaces they may have never entered, through multiple transfer steps [22,24,25,26,27,28,29]. Similarly, non-self DNA, meaning DNA not originating from an item’s owner, can be present on personal and commonly used objects, originating from both unknown sources or from close associates of the items’ owners, depending on their history of use [27,30,31]. Touch DNA can serve as a crucial evidentiary element due to the skin’s primary role as a medium of interaction between the body and the surroundings. Hands in particular can be the vector of indirect DNA transfer, as well as other touched items, leading to the recovery of complex DNA profiles [23,32,33,34,35,36,37]. Extensive research exists on touch DNA transfer probabilities based on various mechanisms and factors that may affect transfer events [10,12,13,14,27,30,32,35,38,39,40,41]. However, more data are needed on the prevalence and composition of bgDNA in different scenarios as not all background DNA is created equal. If an alleged crime were to happen in a public space, the presence of unidentified DNA could potentially complicate the statistical analysis of the evidence. However, if a POI is identified as a contributor to the trace, this will carry greater significance during the assessment at the activity level. On the contrary, the evidential value may decrease when multiple people, such as the victim and the POI, habitually occupy the same spaces and/or have access to the same objects. Thus, POI’s presence is expected to be found to a certain extent. To our knowledge, a few studies researched the prevalence, makeup, and accumulation of bgDNA in both public and private locations and even fewer delved into the source of non-self DNA recovered on individuals’ belongings, either as a “baseline” or indirectly transferred during an activity [16,17,36,42,43,44,45].

In light of these considerations and per the suggestions of the international scientific community on the experimental reproduction of casework conditions [5,6,46], the present work is part of a project aimed at DNA evidence evaluation at the activity level. The prompt for this research came from a real-life case, where our laboratory was tasked with performing a DNA analysis and evidence evaluation at the sub-source level only. However, given the interesting circumstances, its in-house replication was set up to investigate the TPPR of touch DNA traces on credit cards. The details are extensively described in Onofri et al. [47]; however, the key aspects are the following: During sick leave, an individual (O) notices unauthorised cash withdrawals from their credit card. The credit card had been left in a private locker in their office, which was shared with a co-worker (POI) who is suspected of using the card. POI denies using O’s card and states that O frequently left the card on POI and other co-workers’ desks. Our previous work analysed differences in the qualitative and quantitative characteristics of touch DNA traces, whether directly deposited or secondarily transferred. However, of particular interest was the detection of non-prevalent DNA in a large number of samples (86.4%). These findings were unsurprising due to a number of reasons. Previously published data indicate that DNA of an unknown origin is virtually ubiquitous and can be found even on personal belongings [25,30,33,36,42,48,49,50,51,52,53]. The experiment aimed to reflect realistic casework conditions; thus, the substrates employed in the study were not sterilised and participants shared office spaces freely. Furthermore, the items investigated, meaning credit cards, despite being highly personal items, are used in various situations, potentially accumulating foreign DNA (handling by shop clerks, POS devices, ATMs, etc).

This information prompted a more in-depth investigation into the prevalence, composition, and origin of bgDNA on credit cards of individuals who habitually share office space to assess the following:

1. The “baseline” prevalence and composition of bgDNA on the substrates involved in the transfer study: credit cards and personal office desks;
2. Amount and quality of non-self DNA picked up and transferred by the hands during direct contact with objects while staying in shared spaces;
3. Amount and quality of non-self DNA that, after deposition onto a desk surface, can be transferred to a credit card, through object-to-object contact.

These evaluations would aid in determining the surfaces and transfer routes that contribute the most to bgDNA accumulation and composition. The ultimate aim of this study was to obtain probability values for POI being part of the bgDNA composition if POI is a close associate of O. Therefore, the present study was articulated in three parts: Part A) The statistical re-evaluation of the traces resulting from the realistic direct and secondary transfer simulations from the previous study. Part B) The evaluation of the “baseline” prevalence and composition of bgDNA on credit cards and personal desks (as per the above point, point 1). Part C) Secondary or higher degree transfer of foreign DNA (as per the above points, points 2 and 3).

## 2. Results

For continuity purposes, the abbreviations used in [47] are maintained. The owner of the item or donor of a trace is designated as “O”. The participant acting as the person of interest is labelled as “POI” (only present in Part A). The individual identified as an additional contributor to a trace, other than POI and O (Part A) or solely O (Parts B and C), is referred to as the Other Contributor (“OC”). Possible additional contributors to the traces have been looked for amongst the individuals closely associated with the participants; therefore, OC refers to either the participants’ co-workers or to their co-inhabiting partners. When an OC is identified, their relation to the participants is specified.

### 2.1. Part A—Statistical Re-Evaluation of the DNA Traces from the Realistic Transfer Scenarios

The 22 touch DNA samples produced in the previous research underwent a statistical re-analysis to identify additional contributors amongst those who worked and resided closely with the participants. On initial re-evaluation, some samples were better explained by the presence of a greater number of contributors. In total, 91% of samples (20 out of 22) showed non-prevalent DNA. All 11 direct transfer traces exhibited at least one additional contributor besides O and POI, whereas only 2 out of 11 secondary transfer samples were explained solely by O and POI. The difference in the number of contributors observed between the two transfer mechanisms is significant (Student’s *t*-test, *p*-value = 0.011).

An additional individual (OC), besides O and POI, was identified as a contributor to a total of 8 traces. The results are presented in Table 1.

For the sake of brevity, Table 1 displays log10LRϕ values only for the identified OC, calculated by conditioning both the prosecution and the defence hypotheses, Hp and Hd, respectively, on O and POI. Mixture proportion values are those obtained under the hypothesis where O, POI, and OC are all present. The profile completeness of the OC was calculated based on both their complete autosomal genotype and their unique alleles (not shared with O and POI).

In the direct transfer scenario, an additional contributor (OC) was identified in five traces. Among these traces, a co-worker contributed to two traces, while a co-habiting partner contributed to the remaining three. The relative mixture proportion for OC ranged from 0.1 to 0.29, corresponding to DNA amounts ranging from 0.215 ng to 0.533 ng. The OC (Husband A) contributed slightly more to the composition of the trace in sample D8. The findings of the direct transfer scenario provided strong to extremely strong support for identifying the participants’ associates as contributors to the traces, as per conventional and exhaustive LRϕ values. Conservative LRϕ was slightly weaker, as expected.

An individual closely associated with the participants was identified as a contributor to three of the secondary transfer traces, with a co-worker contributing to two samples and Husband A contributing to the remaining trace. The relative contributions appear to be generally higher, with a mixture proportion for OC ranging between 0.19 and 0.5, corresponding to 0.149 and 0.338 ng. Log10LRϕ were sensibly higher than those observed in the direct transfer cases.

In summary, the following was observed in the realistic casework scenario:

- A co-worker was included as an additional contributor (log10LRϕ > 1) in 18.2% of the traces (4 out of 22), meaning 2 out of 11 direct transfer traces (18.2%) and 2 out of 11 secondary transfer traces (18.2%). A log10LRϕ > 6 was observed in 13.6% of the traces (3 out of 22), meaning 1 out of 11 direct transfer samples (9.1%) and 2 out of 11 secondary transfer samples (18.2%);
- A partner was included as an additional contributor (log10LRϕ > 1) in 18.2% of the traces (4 out of 22), meaning 3 out of 11 direct transfer traces (27.3%) and 1 out of 11 secondary transfer traces (9.1%). A log10LRϕ > 6 was observed in 9.1% of the traces (2 out of 22), equally distributed between the two sample groups. In one trace (4.6% of the samples), the OC was the major contributor;
- In total, 91% of the samples contained unexplained, foreign alleles;
- Lastly, the amount of DNA (in ng) contributed by OC and unknown sources was regarded separately. There was no significant difference in OC’s contribution between the two transfer mechanisms (Student’s *t*-test, *p*-value = 0.34), nor was there a significant difference in the contribution from unknown sources (Student’s *t*-test, *p*-value = 0.07). However, there was a significant difference in the overall non-prevalent DNA (Student’s *t*-test, *p*-value = 0.035), which may indicate a combined effect of two factors.

### 2.2. Part B—Participant’s Credit Cards and Office Desks Control Samples

To investigate potential sources of foreign DNA resulting from shared spaces, the study examined surfaces and items that were included in the previous study. The study analysed one frequently used card per participant (N = 4) and the surface of their desks (N = 4). The items were assigned codes according to their owner, meaning “Card A” to “Card D” and “Desk A” to “Desk D”, respectively. The results are shown in Table 2, which displays log10LRϕ values only for the identified OC, calculated by conditioning both Hp and Hd solely on O. Mixture proportion values were obtained under the hypothesis where O and OC are both present. Profile completeness for the OC is calculated on the generated consensus profile based on both their complete autosomal genotype and unique alleles (not shared with O).

Although the yield of DNA from credit cards was lower than that from office desks, the former generally contained a greater amount of foreign DNA. Notably, despite yielding the second lowest DNA quantity, two OCs were identified with Participant A’s card (Card A). One of the individuals was a colleague (Participant B) who works in the closest contact with Participant A, and the other was Husband A. Participant B’s exhaustive log10LRϕ was greater than 6, while the evidence moderately supported Husband A’s contribution. In this case, mixture proportions were those calculated under the hypothesis where O, Participant B, and Husband A were all contributors to the trace. Participant B and Husband A contributed slightly more than the owner of the card. This unexpected observation is further elaborated on in Section 3.2.2. In all card control traces, at least one unknown contributor was observed except in the case of Card A. A larger quantity of DNA was recovered from the office desks. The majority of the touch DNA traces recovered belonged to the owners of the desks (mixture proportion ranging from 0.95 to 0.98), with the exception of Desk B, which had an additional contributor (Participant C) with an exhaustive log10LRϕ equal to 15.89. Participant C contributed to 28% of the trace and had a profile completeness of 78%, as calculated based on their unique alleles.

In summary, the control samples showed the following:

- Cards: partners and co-workers of the participants were identified as contributors (log10LRϕ > 1) to 25% of the samples (1 out of 4) and they showed a greater contribution to the trace than O. Only for the co-worker, log10LRϕ was greater than 6;
- Desks: a co-worker was included as a contributor to 25% of traces (3 out of 4) with log10LRϕ > 6;
- In total, 75% of the reference samples contained unexplained, foreign alleles.

### 2.3. Part C—Secondary or Higher Degree Transfer of Foreign DNA

One of the objectives of this study was to identify the primary route of foreign DNA deposition in shared environments, as outlined in Section 4.3. Participants were requested to handle, without gloves, four sterile credit cards each (N = 16) to evaluate foreign alleles picked up and subsequently deposited by the participants’ hands. Additionally, the participants’ propensity to release self DNA by touch, defined as the shedder status, was assessed [54,55,56,57,58,59,60]. Intra- and inter-individual variations were investigated to understand how they relate to non-self DNA transfer [32,34,37,45,54,61,62,63]. Additionally, participants were instructed to touch a previously sterilised area on their desks during the workday. Afterwards, a sterile credit card per participant was lightly rubbed on the desk to study the transfer of non-self alleles from one surface to another (N = 4). Samples were labelled according to the mode of transfer, followed by the letter designating the participant (from A to D), and the card number. Direct transfer traces were coded as “Direct (Code of the Participant).1” to “Direct (Code of the Participant).4”. The samples from the item-to-item scenario were assigned the code “Secondary A” to “Secondary D” since only one card per participant was used. For continuity purposes, the participant donor of the trace is labelled as “O”. During this time, participants were allowed to carry on with regular day-to-day activities. They were, however, forbidden from wearing gloves.

**Figure 1:**
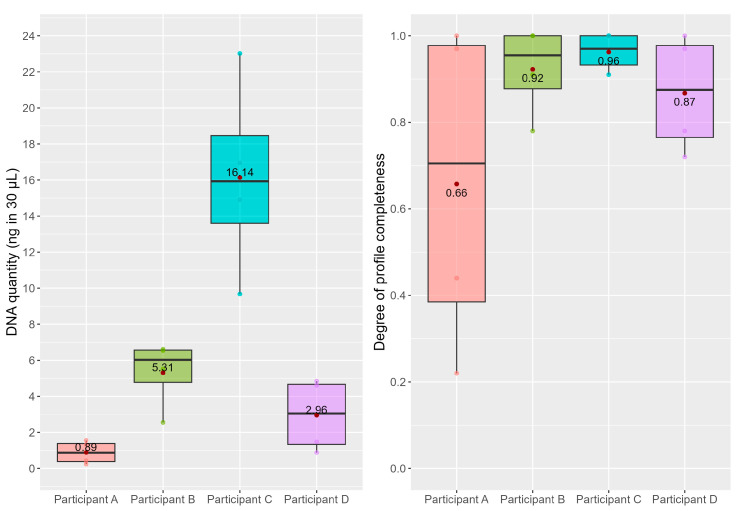
Graphical representation of the results of Part B of this study, concerning the direct handling of sterile credit cards by the participants for the evaluation of inter- and intra-individual variation in shedding propensity. On the left, the DNA amount (ng in 30 μL) deposited on the cards is reported, while on the right, the respective profile completeness of the donor of the trace (O) is reported. Mean values are marked as red dots and their numerical value is reported in the boxplots.

To assess the participants’ shedding propensity, two factors were evaluated: the total amount of DNA deposited during the handling of the cards and the completeness of the resulting O’s. The obtained results are shown in Figure 1. Based on quantitation data (Table 3), Participant C deposited the highest amount of DNA, with a mean of 16.14 ng in 30 μL, followed by Participant B (mean = 5.31 ng/30 μL), Participant D (mean = 2.96 ng/30 μL), and lastly Participant A (mean = 0.89 ng/30 μL). To check whether the observed inter-individual differences were statistically significant, the Wilcoxon–Mann–Whitney U-test was conducted (alpha = 0.05). There was no significant difference observed between Participants A and D, and Participants B and D (*p*-value = 2 and *p*-value = 0.1143, respectively). As shown in Figure 1, Participant D shed an intermediate quantity between Participants A and B. Intra-individual variability was estimated by calculating the standard deviation in the deposited DNA amount for each participant. Participant C showed the greatest variability in terms of shedding propensity (sd = 2.76) followed by Participant D (sd = 1.03) and B (sd = 0.97). Participant A was the most consistent in terms of deposited DNA (sd = 0.32). These findings support the classification of shedders according to three categories, Poor, Intermediate, and Good shedders, as previously proposed [47,56,63,64,65,66]. While Participant C was confirmed to be the only Good shedder of the volunteers, the other participants were in part re-evaluated. In particular, Participant B could be classified as an Intermediate shedder along with Participant D, while Participant A turned out to be not an Intermediate shedder, as previously classified, but a substantially consistent Poor shedder. 

The evaluation of the participants’ profile completeness did not reflect quantitation results. As expected, a larger quantity of DNA deposited resulted in a more complete DNA profile, while lower amounts led to an incomplete genotype. Indeed, as shown by Figure 1, Participant A exhibits a wider range of profile completeness (sd = 0.39) compared to the smaller variance observed in terms of the DNA amount. No significant difference among participants in the completeness of their profiles (calculated based on the consensus profile) was observed. Therefore, evaluations based on deposited DNA quantities may be a more solid criterion for assigning an individual their shedder status.

As previously noted [47], DNA traces resulting from direct handling generated a larger quantity of DNA, displaying a more comprehensive O profile relative to traces obtained from a secondary transfer scenario, alongside a greater number of non-self alleles and contributors. Results are reported in Table 3. For purposes of brevity, the table displays log10LRϕ values only for the identified OC, calculated by conditioning both Hp and Hd solely on O, and mixture proportion values obtained under Hp where O and OC are both present. Profile completeness for the OC was calculated on the generated consensus profile based on both their complete autosomal genotype and unique alleles (not shared with O).

Only one trace (Direct A.4) presented one contributor, reporting an extremely incomplete profile of the donor; therefore, it was deemed not comparable. The remaining 15 traces were multi-contributor mixtures. In total, 81.25% of the samples (13 out of 16) presented two contributors, while two traces were better explained by three contributors and the remaining one by four. Non-self alleles were counted based on the traces’ consensus profile, revealing that 87.5% of the samples (14 out of 16) presented alleles of an unknown origin (Table 3). A weak/moderate negative correlation was observed between the amount of deposited DNA, and thus the shedding status, and the number of foreign alleles detected (Pearson’s r = −0.307). No OCs were identified with these traces; therefore, no further statistical evaluation was performed.

The amount of self and non-self DNA recovered from the cards after item-to-item contact (through the office desk surface) was generally lower than that yielded with direct transfer traces. In total, 75% of the samples (three out of four) presented unknown alleles. The only trace with zero non-self alleles (Secondary A) was explained solely by O, who reported an extremely incomplete profile. Therefore, it was deemed not comparable. The highest amount of transferred self and non-self genetic material was observed for the trace Secondary D. A co-worker (Participant D) was identified as an additional contributor (OC) to the traces of Participants B and C (Secondary B and Secondary C), with a relative contribution of 0.055 ng (mix. prop. = 0.21) and 0.117 ng (mix. prop. = 0.08), respectively.

To summarise, in the transfer simulation samples, the following was observed:

- Direct manipulation: no additional contributor was identified in these traces. However, unexplained, foreign alleles were present in 81.25% of the traces (14 out of 16);
- Secondary transfer: unexplained, foreign alleles were present in 75% of the samples (three out of four). A co-worker was included as a contributor to 50% of the traces (two out of four) with a log10LRϕ greater than 6; however, the donor of the trace was the major contributor.

## 3. Discussion

Before discussing the results obtained, it should be noted that, in general, higher amounts of DNA were recovered from the credit cards analysed in Parts B and C compared to the previous study. This increased yield could be attributed to the recovery technique used. In the previous study, only the tape-lifting technique was employed, while in the present study, the substrates were sampled by using the double-swabbing technique followed by tape-lifting on the same area. This combined technique may potentially increase the yield. However, a study on the recovery efficiency of these two sampling methods from a plastic support is needed.

### 3.1. Part A

The presence of non-prevalent DNA observed in [47] is confirmed through the detection of foreign alleles in 91% of the samples. These results are consistent with previous findings [30,33,36,48,49,50]. Research conducted on the accumulation and transfer of touch DNA traces shows that personal items may contain not only their owner’s DNA but also genetic materials from the owner’s close associates, such as co-inhabiting partners and co-workers [27,30,31]. In fact, the occasional or habitual presence of an individual in an environment is sufficient for the release of their DNA, through shedding of skin cells, speaking, or touching the surroundings [18,19,20,21,22,23,32,33,34,35,36,37]. Additionally, through multiple transfer steps, an individual’s genetic material may be found on another person’s belongings or in places they have never visited [22,24,26,27,28,29]. Considering the realistic casework conditions, the possible sources and routes of non-prevalent DNA deposition may be various. First, the substrates involved in the study were not sterilised, determining the detection of bgDNA previously accumulated on the credit cards. Secondly, the hands of the participants had not been washed nor sterilised prior to the direct handling experiment, possibly acting as a vector for non-self DNA transfer from the surroundings to the cards. Thirdly, the participants’ desks had not been sterilised, possibly resulting in the transfer of non-self DNA accumulated there onto the credit cards during the secondary transfer simulations. Therefore, the 22 traces from [47] were statistically re-analysed to identify probable additional contributors to the non-prevalent DNA, initially referred to as background DNA of an unknown origin. In the present work, both co-workers and co-inhabiting partners of the participants were identified as contributors to the non-prevalent portion of the traces. In 13.6% of the traces from the direct transfer scenario and in 9.1% of the samples from the secondary transfer simulations, said individuals were identified as additional contributors with a log10LRϕ > 6. Furthermore, in 4.6% of the cases, a close associate (co-inhabiting partner) was identified as the major contributor.

After establishing that a portion of the non-prevalent DNA may derive from individuals closely associated with the owners of the items, the quantity of DNA attributable to OC’s contribution and to unknown sources was investigated separately. Differences in terms of non-prevalent contributions to the trace due to the mode of transfer had been previously observed [47]. It was initially hypothesised that a significant amount of foreign DNA may have been deposited by the participants during the direct handling experiment, while a smaller quantity would have been transferred in the secondary transfer scenario. However, it was also noted that log10LRϕ values for the additional contributors (OC) found in the secondary transfer traces were higher than those observed in the direct transfer cases (Table 3). This could be attributed to the fact that, in the direct transfer samples, the POI’s freshly deposited material may partially mask previous contributors and remove some of the genetic material that had accumulated onto the card prior to the manipulation experiment. In the secondary transfer scenario, the rubbing of the cards onto the desk surfaces may have also caused a partial loss of genetic material already present on the cards, while the transfer from the desks to the cards did not mask the presence of OC.

In a realistic casework situation, it should be taken into account that individuals who work and live in close proximity to O could significantly contribute to the makeup of touch DNA traces recovered from personal items. In cases where POI and O are closely associated, it would be extremely difficult to distinguish whether the recovery of POI’s DNA is the result of accumulation over time—and thus, bgDNA composition—or of the disputed activity. Additionally, even if POI’s DNA was transferred during the activity in question, their contribution may sum up to earlier “innocent” deposits. If possible, the sampling and analysis of the item’s region adjacent to the area of interest may aid in such evaluations [67,68]. However, in this case, inferring probabilistic values for POI’s presence appears to be more fitting. Considering the criteria employed in the previous study, meaning log10LRϕ > 6 and major contribution, it was necessary to investigate the probability of POI being a contributor to a trace due to their presence as part of the background DNA of the items—and not due to either disputed activity (direct transfer or secondary transfer of their DNA)—if not even secondarily transferred by POI’s hands during the disputed action.

### 3.2. Parts B and C

Given the design of the transfer study, it was crucial to comprehend the deposition and transfer of non-self DNA onto personal items and surfaces in shared spaces, as well as the “baseline” composition and abundance of background DNA on the items before the event in question. Additionally, part of the foreign material may have been transferred during the transfer experiments.

In summary, non-prevalent DNA on the card could be the following:

- Part of the background DNA of the items in question;
- Present on POI’s hands and then tranferred during the manipulation of the cards;
- Present on POI’s desk and then transferred during the rubbing of the cards on the desks.

Part B of the experiment aimed to establish “baseline” values of prevalence and composition of background DNA on the surfaces involved in the transfer study, namely credit cards and office desks. The credit cards yielded a lower amount of material than the personal desks while presenting a higher proportion of foreign DNA, which is expected due to the characteristics and nature of the substrates. Card A produced the most interesting result. Both the co-inhabiting partner and a co-worker were identified as contributors to the card. Husband A’s contribution could be more easily expected. Instead, despite Participant B being the colleague who works in closest contact with Participant A, their presence as a major contributor was surprising. Upon investigation, Participant A stated that the day before the experiment they asked Participant B to withdraw cash for them from the ATM, explaining the results obtained. These findings reflect how an item’s background DNA composition may vary depending on its history of use [16,48].

Office desks have been found to produce a greater amount of DNA due to frequent contact with participants’ hands and forearms throughout the day. DNA can also accumulate on these surfaces through shedding and saliva droplet deposition, unlike credit cards. The higher yields for Participants A and D compared to B and C can be accounted for by the extensive period of time that the two participants usually spent at their desks. Participant C’s presence as the second most prominent contributor to Desk B is in line with previous findings on DNA deposition in shared environments [17,22,29,32,39,43,69]. The remaining unknown alleles could be attributable to the cleaning crew but given the design of the experiment and that foreign DNA accounted for only minor contributions, such evaluation was not carried out.

The amount of genetic material retrieved from the two substrates investigated and relative contributions from both known and unknown sources varied greatly depending on the type of item and their history, frequency, and mode of use. Therefore, more studies are needed to understand how the strength of the evidence changes according to these factors in conjunction with the relevance and significance of an item based on the case-specific circumstances. In this case, it was essential to understand the bgDNA composition on both office desks and credit cards since they are crucial to the disputed activities. The recovery of a co-worker’s genetic material from O’s personal office desk may not be significant since their presence on the surface has been observed and is expected to a certain degree. At the same time, the presence of said co-worker on O’s credit card is not attributable to bgDNA composition due to co-working but to a prior use, supporting the findings in [47]. Moreover, the analysis of different items, such as a computer keyboard or phone, may be inconsequential for evidence evaluation at the activity level.

In Part C, the participants’ shedder status was assessed and intra- and inter-individual variations were evaluated. Additionally, in accordance with the consideration outlined above regarding potential pathways for foreign DNA transfer, the non-self DNA was evaluated in two transfer mechanisms:

- Direct deposition: non-self DNA accumulated during the workday on the participants’ hands and deposited through direct handling of credit cards;
- Secondary transfer: non-self DNA present on the participants’ hands, deposited onto their office desks during the workday, and subsequently transferred on credit cards rubbed on the desks.

#### 3.2.1. Direct Deposition

The observed intra- and inter-individual variability in terms of quantity of DNA deposited via touch supports in part the findings of [47] in which the same participants were assessed using a different approach. In the previous study, the participants’ shedder status was assessed at four different time intervals after hand washing by holding with both hands a 15 mL falcon tube for 30 s. In [47], two characteristics were evaluated, DNA yield and profile completeness. Instead, the present study aimed at assessing shedding propensity by asking the participants to handle a credit card for 30 s during the course of a regular workday.

Participant B, who was originally classified as a Poor shedder, now showed a greater amount of deposited DNA than Participants A and D (Figure 1), previously designated as Intermediate shedders. Conversely, Participant A shed the least amount of genetic material compared to the rest of the group (Figure 1). The difference in the shedder categorisation of Participant B between the two studies could be initially attributed to changes regarding the participant between the two experiments. However, there was no alteration in the participant’s skin condition, habits, or activities performed in the workplace. The two experiments’ study design was then considered, since a number of factors could have contributed to an increased DNA deposition by the participants’ hands. As mentioned above, the shedder status was assessed in [47] by evaluating the touch DNA traces deposited at different time intervals after hand washing and in the present one, the assessment was carried out during the workday. Hand washing has been shown to reduce the amount of self DNA deposited during touching [47,70] and the longer the time elapsed since a hand wash, the greater the amount of DNA deposited [47,56]. Therefore, in general, a greater amount of DNA could have been deposited by the participants during the workday, in the second experiment. Additionally, the type of contact and surface area involved differed between studies. In the first one [47], the body of a 15 mL falcon tube was touched by the participants’ hands, including the palmar surface. In contrast, in the present study, credit cards were used, providing a smaller surface of contact that was touched mainly by the fingertips. However, fingertips have been found to have a higher shedding propensity than the palm [71]; thus, the involvement of the palm in the previous study may be negligible. Lastly, in [47], a “static” touch, with no friction, was performed on the object, while the present study saw the participants actively handling the cards, possibly determining the deposition of greater amounts of DNA. The active handling action combined with the increased accumulation of self DNA on the participants’ hands during the workday may be responsible for the observed increase in DNA deposited across all participants. In fact, the difference in study design is true for all, not just for Participant B. Therefore, it is hypothesised that the discrepancy observed for Participant B may be due to an intrinsic individual characteristic. In fact, Participant B may need more time than the other participants to progressively build up self DNA on their hands, while in general releasing more material than Participants A and D at a random sampling time.

The observed variability in shedding propensity among participants supports the shedder classification according to three categories, as previously proposed [47,56,58,63,64,65,66]. In fact, the Good and Poor shedders were easily identified (Participants C and A, respectively). However, the variation in amounts of shed DNA denoted a range of subtle differences, especially between Participants B and D (Intermediate shedders), and between them and Participant A. Therefore, a more extensive investigation on intra- and inter-individual variability would be needed to better assess the ranges of an individual’s propensity to deposit self DNA and to more accurately establish criteria for assigning an individual to the most fitting shedder category.

As discussed in Section 2.3, the quantitation results were not reflected by profile completeness values. A higher amount of deposited genetic material corresponds to a more complete and less variable profile, whereas a smaller quantity of DNA results in more incomplete profiles. Participants C, B, and D exhibited higher mean values but greater variability in terms of shed DNA, and the traces had a relatively smaller variability in terms of profile completeness. However, Participant A exhibited a wider range of profile completeness compared to the smaller variance observed in terms of the DNA amount. This discrepancy may be attributed to the strictness of the consensus profile method, also considering that variations in terms of alleles present in the EPG could stem from PCR amplification stochastic errors given the minute quantities of template material [72,73]. Therefore, in contrast with the observations by Lee et al. [65], it is suggested that the shedder status be evaluated by taking into consideration DNA quantitation data, which appear to be more informative and more discriminating among individuals.

Previous studies reported a correlation between a Poor shedding status and a tendency to pick up and deposit higher amounts of non-self alleles [45,54,61,62,63]. In this study, we observed that there is a moderate negative correlation between the amount of directly deposited DNA and the number of foreign alleles detected (Pearson’s r = −0.307). This finding can be explained by the fact that the greater the amount of self DNA deposited, the more non-self DNA present in smaller quantities is being masked [33]. This effect is not strictly limited to the masking on non-self alleles but may also take place when multiple individuals touch the same item, leading to varying degrees of contribution due not only to the history of use of the item, but also to the individuals’ shedding propensity [34,37,48,69,74,75]. An individual with a higher shedding propensity may mask the presence of a poorer shedder, while, at the same time, a Poor shedder may not deposit detectable traces during the use of an item. The shedder status of an individual may also influence the probability of a secondary or higher degree transfer of their DNA [33,59,63]. Therefore, to evaluate touch DNA traces at the activity level in an informed and comprehensive manner, it is essential to study intra- and inter-individual differences in shedding propensity, along with understanding how these differences affect the detection of self and non-self DNA. Additionally, on a technical level, a single-cell analysis may be employed for the successful detection and typing of minute contributions of cellular material but not cell-free DNA [2,76].

#### 3.2.2. Secondary Transfer through Item-to-Item Contact

The DNA yield from the traces obtained in the secondary transfer scenario was generally lower. However, three out of four traces (75%) had two additional contributors besides the participant who deposited them (O). These results can be explained by the progressive accumulation of foreign genetic material on the desk surface. In fact, a co-worker (Participant D) was identified (log10LRϕ > 6) as a contributor to two out of four samples, namely Secondary B and Secondary C. However, in both cases, Participant D was not the major contributor to the trace. It is worth noting that Participants B and C’s working stations are close to Participant D’s desk. Therefore, given the data available on DNA deposition and its secondary or higher degree transfer in shared environments [17,22,29,32,39,43,62,69], said contribution is not surprising. This was also observed in the control samples for Desk B, where Participant C was identified as a contributor. Part B results explain the realistic casework scenarios’ traces S3 and S4 (Table 1) [47], in which Participant B was identified as an additional contributor. In fact, these traces resulted from Participant D’s card being rubbed on Participant C’s desk and vice versa, with no intervention of Participant B; thus, they should have not been identified as a contributor. Therefore, Participant B’s DNA could have been present on the office desks of the other two participants, as it was the case in the traces Secondary B and Secondary C. In the future, it may be useful to investigate the probability of POI’s DNA transfer through other individuals’ desks or other surfaces present in the office.

## 4. Materials and Methods

This study was approved by the Ethics Committee of the University of Perugia (protocol code 92184, 14 March 2023). The participant cohort comprised four laboratory staff members who had previously taken part in the touch DNA transfer mechanisms study [47] and they were assigned the same code (Participant A-D). Given the aim of the current study, which is to investigate the prevalence, composition, and routes of the accumulation of bgDNA on the substrates involved in [47], reference samples were collected from the close associates of the participants. After informed consent was provided, buccal swabs were taken from the co-inhabiting partners of Participants A and C and were assigned the codes Husband A and Husband C, respectively. A staff member, who works in a dedicated office but occasionally transits through the shared spaces, agreed to the use of their genotype (already present in the laboratory exclusion database) as a reference and he was assigned the code Coworker 1. To minimise the probability of contamination events, the volunteers were forbidden access to laboratory spaces after their thorough sterilisation. The sample collection and analysis were conducted by personnel who did not share spaces with the participants or take part in this study.

In the previous study on touch DNA transfer mechanisms, the source and composition of non-prevalent DNA were not examined and the foreign contribution was broadly described as “Unknown”. Taking into consideration the realistic casework conditions, which included the use of unsterilised items, and the transfer mechanisms simulated, either direct manipulation by the POI or secondary transfer via the POI’s desk, the possible origins and routes of transfer of the foreign DNA could be various. Hence, a detailed study of each factor involved in the deposition of non-prevalent DNA was essential, in accordance with the objectives set out in Section 1. Therefore, the conducted research aimed to provide a thorough investigation of each aspect involved in the transfer of foreign DNA.

The present study was articulated in three parts.

### 4.1. Part A—Statistical Re-Evaluation of the DNA Traces from the Realistic Transfer Scenarios

To begin with, it was necessary to identify possible additional contributors to the traces produced in [47]. The EPGs of the 22 credit cards were first manually compared with the reference genotypes of the individuals who, given their close association with the participants, were likely to be present. For instance, Husband A’s DNA may be present on his spouse’s belongings, but it is unlikely that Husband C’s DNA is present on a card owned by Participant A and handled by Participant B. The probable contributor’s profile completeness was manually assessed, taking into account both all the individual’s alleles and only their unique alleles (not shared by either O or POI). If the associate’s profile matched the trace in at least 10 loci, in accordance with Ge.F.I. guidelines [77], then the Likelihood Ratio calculations at the sub-source level (LRϕ) were conducted using EuroForMix v 4.0.1 [78] and EFMex [79], applying the same parameters used in [47].

In the previous study, O’s presence was expected, due to the ownership of the card; therefore, the presence of POI as a contributor was disputed by conditioning the LRϕ calculations on O. In the present study, it was necessary to evaluate the presence of an additional contributor, OC, based on a double criterion; thus, the conventional (Maximum Likelihood Estimation-based) and conservative LRϕ for OC were calculated twice. Firstly, Hp and Hd were conditioned solely on the card owner (O), in order to only dispute the OC presence, as if OC was another POI. Secondly, OC’s LRϕ was calculated by conditioning both hypotheses on both O and the POI, and thus accepting POI’s presence on the cards as true. An additional evaluation was conducted by using EFMex, for the comparison of multiple POIs to a trace according to what is known as the exhaustive method [80,81], by conditioning solely on O. The exhaustive LRϕ value was obtained. The tested associates with conventional LRϕ values greater than 1 were accepted as contributors to the trace, labelled OC.

The mixture proportions examined here are those obtained under the Hp where O, POI, and OC are all present. To determine OC relative contribution in terms of DNA quantity (ng), the mixture proportion was multiplied by the total DNA quantity (ng in 30 μL). The number of unknown alleles, which did not belong to O, POI, or OC, was also calculated.

### 4.2. Part B—Participant’s Credit Cards and Office Desks Control Samples

As per the previous study’s design, two different transfer mechanisms were simulated—primary, in which the participant designed as POI directly handled the cards and secondary, by rubbing the cards onto the POI’s desk. Neither the cards nor the desks had been previously sterilised. Therefore, the “baseline” abundance and composition of bgDNA on the items involved in this study were examined by analysing one regularly used credit card per participant (N = 4) and their office desks (N = 4). The cards were sampled in their entirety while the desk collection was limited to a 10 by 10 cm square of the desk’s surface that is subjected to the most contact with the occupant.

Card and desk control samples were assigned codes according to their owner, meaning “Card A” to “Card D” and “Desk A” to “Desk D”, respectively.

### 4.3. Part C—Secondary or Higher Degree Transfer of Foreign DNA

Taking into account the transfer study’s design, two potential routes for the deposition of non-prevalent DNA have been hypothesised. In the direct transfer experiment, the participants’ hands may have picked up non-self DNA while interacting with the environment, which was then transferred while handling the card. In this case, the foreign DNA must have resulted from secondary or higher degree transfer.

In the secondary transfer scenario, the foreign DNA may have been picked up by the participants’ hands and then deposited on their desks or it may have been deposited by their co-workers during the regular use of the office spaces (through shedding, saliva droplets, touching, etc.). In either case, the non-self DNA deposited on the personal desks could have been transferred to the cards, during the item-to-item contact, as a secondary or higher degree transfer. Additionally, previous research has indicated a potential link between the shedder status and the amount of non-self DNA picked up and deposited by a person’s hands [32,34,37,45,54,61,62,63]. Specifically, it was observed that individuals with a poorer shedding propensity had a greater likelihood of picking up and depositing detectable amounts of foreign DNA [45,54,61,62,63].

To investigate the quantity and quality of foreign DNA that can be picked up and directly deposited by the participants’ hands during their workday, as well as to gather more information about their shedder status, the participants were asked to handle, without gloves, four credit cards each for 30 s (N = 16) on different days. To study the characteristics of non-self DNA that accumulates on participants’ desks during the workday and consequently transfers onto the cards, participants were instructed to touch a designated 10 × 10 cm area of their desk. Following this, a card was gently rubbed on the same spot for 30 s (N = 4). Samples were labelled according to the mode of transfer, followed by the participant, and the card number. For instance, the traces resulting from Participant A directly handling the card were “Direct A.1” to “Direct A.4”. The samples from the item-to-item scenario were assigned the code “Secondary A” to “Secondary D” since only one card per participant was used. For continuity purposes, the participant donor of the trace is labelled as “O” as well.

During the experiment, participants were instructed to carry on with regular everyday activities; however, they were forbidden from wearing gloves. To reduce any potential DNA contamination from previous deposits, the cards utilized in this phase of the experiment were sterilised beforehand in a bleach bath for 10 min and then irradiated with UV lights 30 min per side. The desks were sterilised with bleach.

### 4.4. Laboratory Processing

No laboratory work was required for Part A of the experimental setup. Due to discordant previous findings regarding the most effective method for touch DNA collection on these types of substrates [82,83,84,85,86], a combination of double-swabbing and tape-lifting techniques [31,71] was used to collect samples from the items (cards and desks) in Parts B and C. All samples were extracted by using the phenol–chloroform organic method [87]. The two cotton swabs (MEUS srl, Piove di Sacco, Italy) were co-extracted, whilst the respective tape was extracted separately. The swabs were resuspended using 30 μL of sterile water, and subsequently, the resulting eluate was used to resuspend the pellet of the corresponding tape to obtain a single extract per item. Quantification was carried out using the PowerQuant^®^ System (Promega, Madison, WI, USA) [88,89] on the Applied Biosystems™ 7500 Real-Time PCR instrument (Thermo Fisher Scientific, Waltham, MA, USA). The samples were amplified as is or, if necessary, diluted to satisfy the PCR kit requirements. A DNA concentration threshold was not applied; thus, all DNA extracts, including those with a scarce amount of template DNA, were processed. PCR amplification was performed in duplicates with the PowerPlex^®^ ESX17 Fast kit (Promega, Madison, WI, USA), according to the product’s protocol [90,91] on the Mastercycler^®^ ep thermal cycler (Eppendorf, Hamburg, Germany). Capillary electrophoresis (CE) was carried out on the Applied Biosystems^TM^ SeqStudio^TM^ Genetic Analyzer (Thermo Fisher Scientific, Waltham, MA, USA). Run conditions were the following: injection time, 7 s; injection voltage, 1200 V; run time, 1440 s; run voltage, 9000 V. The resulting CE data were analysed with GeneMapper^®^ ID-X v 1.6 (Thermo Fisher Scientific, Waltham, MA, USA) and an analytical threshold (AT) of 50 RFU was applied.

### 4.5. Sub-Source LR Calculations and Statistical Analysis

The total DNA amount recovered was calculated by multiplying quantitation results by the resuspension volume (30 μL). The EPGs obtained for the items and cards from Part B and Part C were compared with their owner/donor (O) and with the close associates of the participants, selected based on the same rationale as described in Section 4.1. Conventional, conservative, and exhaustive LRϕ values were computed using EuroForMix and EFMex for both O and the participants’ close associates deemed probable contributors. In this case, either no conditioning was applied or both Hp and Hd were conditioned on O. For conventional LRϕ values greater than 1, the tested associate was accepted as an additional contributor (OC).

The mixture proportions examined here are those obtained under the Hp where both O and OC are present. If no additional contributor is identified, mixture proportions are those under Hp where only O is present. Relative contribution in ng of DNA was calculated by multiplying relative mixture proportions by total DNA quantity.

To be more stringent in further evaluations, profile completeness for O and OC (when present) and the number of foreign alleles were calculated on the consensus profile generated with R. An allele was included in the consensus profile if present in both replicas of the same sample.

An additional statistical analysis was performed using R, and plots were generated by using ggplot2.

## 5. Conclusions

A previous study on touch DNA transfer mechanisms onto credit cards [47] prompted further investigation into the prevalence, composition, and origin of bgDNA on these items. When replicating realistic casework conditions, non-prevalent DNA can be deposited through various routes, before, during, or after the event in question. The evaluation of touch DNA samples can be particularly challenging in cases where multiple individuals share the same spaces, leading to the recovery of traces exhibiting a high complexity in terms of both known and unknown contributions.

The present study demonstrates how an individual’s co-workers and co-inhabiting partners can ordinarily contribute to the traces recovered from their personal belongings. When considering possible transfer mechanisms, hands can pick up a certain amount of non-self material, although not in sufficient quantities to produce a full or partial profile of the foreign source. Conversely, foreign, non-self DNA can accumulate on a surface either through transfer by the owner’s hands over time or via the simple inhabiting of the shared space. This present mechanism appears to be more effective at generating more complete profiles from non-self DNA sources, resulting in comparable results even after subsequent deposition onto an additional surface.

Depending on the case circumstances, different approaches can be employed for bgDNA evaluation on evidence, such as the sampling of areas adjacent to the one of interest. However, this is not always feasible, and a probabilistic approach appears preferrable. In conclusion, a more comprehensive evaluation of touch DNA traces at an activity level requires collecting information not only about DNA transfer probabilities but also about the presence of the person of interest (POI) as part of the ‘baseline’ background DNA of the substrates involved in the investigation. The inferred probabilities can be easily implemented in an ad hoc built Bayesian Network along with other case-tailored information. If transfer and prevalence have been considered so far, other variables must be investigated for more accurate considerations. The time elapsed between the disputed activity and the analysis is often crucial for assessing the values of DNA evidence at the activity level. Studies will be conducted to determine touch DNA persistence on hard plastic surfaces and how the strength of the evidence changes over time. Additionally, the choice of the sampling technique is crucial for optimal DNA yield and for the successful recovery and detection of minute DNA traces. Therefore, it will be necessary to investigate the influence of different recovery methods on the typing results and evidence evaluation.

## Figures and Tables

**Table 1:** Part A results. Statistical re-evaluation of the samples from Onofri et al. [41]. Traces are assigned the same code as in [41], with “D” referring to the Direct transfer simulation and “S” referring to the Secondary transfer scenario, followed by the number of the trace. The table shows the traces for which an additional contributor (Other Contributor, OC) has been identified among the participants’ close associates, meaning co-workers and co-inhabiting partners. The log10LRϕ for OC reported here are those calculated with EuroForMix by conditioning both the prosecution and defence hypotheses, Hp and Hd, on the owner of the card (O) and the person of interest (POI). All three LR calculation methods employed here are reported: conventional (Conv.), conservative (Cons.), and exhaustive (Ex.). The reported OC contribution in terms of mixture proportion (mix. prop.) was that obtained under the hypothesis where O, POI, and OC are all present. The relative contribution of OC in terms of quantity (ng) was calculated by multiplying the mixture proportion by the total DNA quantity yielded for each trace. Profile completeness was calculated based on both all their autosomal alleles (All alleles) and OC’s unique autosomal alleles (Unique alleles). The number of foreign alleles of unknown origin is reported. NoC = number of contributors; Part. = Participant; Husb. = Husband.

Trace	NoC	O	POI	OC	log10LRϕ(OC)			OC Contribution		Profile Completeness OC		Unknown Alleles
					Conv. (MLE)	Cons.	Ex.	Mix. Prop.	ng	AllAlleles	Unique Alleles	
D2	4	Part. A	Part. B	Part. C	8.13	6.38	8.1	0.25	0.53	0.84	0.73	2
D4	4	Part. A	Part. B	Husb. A	5.8	4.01	5.13	0.21	0.22	0.84	0.58	10
D6	4	Part. A	Part. B	Part. C	4.64	2.55	4.3	0.14	0.31	0.94	0.87	11
D8	4	Part. A	Part. C	Husb. A	5.78	4	5.72	0.29	0.27	0.63	0.50	8
D9	4	Part. A	Part. B	Husb. A	7.32	5.61	7.24	0.10	0.23	0.91	0.75	8
S3	3	Part. D	Part. C	Part. B	18.26	16.62	18.26	0.26	0.27	0.97	0.95	1
S4	4	Part. C	Part. D	Part. B	7.92	5.9	7.89	0.15	0.23	0.88	0.79	9
S8	4	Part. A	Part. B	Husb. A	18.79	16.82	18.79	0.50	0.34	1	1	12

**Table 2:** Part B results. For each participant, a regularly used credit card and office desk were sampled in order to analyse the items’ “baseline” levels of background DNA. The log10LRϕ for OC reported here are those calculated with EuroForMix by conditioning both the prosecution and defence hypotheses, Hp and Hd, on the owner of the item (O). All three LR calculation methods employed here are reported: conventional (Conv.), conservative (Cons.), and exhaustive (Ex.). The reported mixture proportions were those obtained under hypothesis where O and OC are both present. The relative contributions in terms of quantity (ng) were calculated by multiplying the mixture proportion by the total DNA quantity yielded for each trace. Profile completeness was calculated based on both all their autosomal alleles (All alleles) and OC’s unique autosomal alleles (Unique alleles). The number of foreign alleles of unknown origin is reported. NoC = number of contributors; Part. = Participant; Husb. = Husband.

Trace	Quantity (ng/30 μL)	NoC	O	OC	log10LRϕ(OC)			Mixture Proportions			Relative Contributions (ng)			OC Unique Alleles	Profile Completeness OC		Unknown Alleles
					Conv. (MLE)	Cons.	Ex.	O	OC	Unk	O	OC	Unk		All Alleles	Unique Alleles	
Card A	1.56	3	Part. A	Part. B	7.57	5.57	6.49	0.28	0.39	-	0.44	0.61	-	22	0.75	0.55	5
				Husb. A	3.3	1.26	2.22		0.33			0.51		12	0.72	0.42	
Card B	0.95	3	Part. B	-	-	-	-	0.85	-	0.14	0.80	-	0.13	-	-	-	4
Card C	12.95	3	Part. C	-	-	-	-	0.82	-	0.18	10.62	-	2.33	-	-	-	7
Card D	3.84	2	Part. D	-	-	-	-	0.95	-	0.05	3.65	-	0.19	-	-	-	0
Desk A	11.93	3	Part. A	-	-	-	-	0.97	-	0.04	11.57	-	0.48	-	-	-	2
Desk B	1.79	3	Part. B	Part. C	12.51	10.71	15.89	0.67	0.28	0.05	1.20	0.50	0.09	23	0.91	0.78	4
Desk C	3.83	2	Part. C	-	-	-	-	0.95	-	0.05	3.64	-	0.19	-	-	-	1
Desk D	8.99	2	Part. D	-	-	-	-	0.98	-	0.02	8.81	-	0.18	-	-	-	0

**Table 3:** Part C results. Touch DNA traces resulting from the participants directly handling the cards and from a secondary transfer through item-to-item contact were investigated for the identification of possible additional contributors (OC). The log10LRϕ for OC reported here are those calculated with EuroForMix by conditioning both the prosecution and defence hypotheses, Hp and Hd, on the owner of the item (O). All three LR calculation methods employed here are reported: conventional (Conv.), conservative (Cons.), and exhaustive (Ex.). The reported mixture proportions were those obtained under hypothesis where O and OC are both present. The relative contributions in terms of quantity (ng) were calculated by multiplying the mixture proportion by the total DNA quantity yielded for each trace. Profile completeness was calculated based on both all their autosomal alleles (All alleles) and OC’s unique autosomal alleles (Unique alleles). The number of foreign alleles of unknown origin is reported. NoC = number of contributors; Part. = Participant; Husb. = Husband; n.c. = not comparable.

Trace	Quantity (ng/30 μL)	NoC	O	OC	log10LRϕ(OC)			Mixture Proportions			Relative Contributions (ng)			OC Unique Alleles	Profile Completeness OC		Unknown Alleles
					Conv. (MLE)	Cons.	Ex.	O	OC	Unk	O	OC	Unk		All Alleles	Unique Alleles	
Direct A.1	1.56	4	Part. A	-	-	-	-	0.75	-	0.25	1.17	-	0.39	-	-	-	11
Direct A.2	0.432	2		-	-	-	-	0.56	-	0.44	0.24	-	0.19	-	-	-	6
Direct A.3	1.32	2		-	-	-	-	0.71	-	0.29	0.93	-	0.39	-	-	-	11
Direct A.4	0.246	1		n.c.	n.c.	n.c.	n.c.	n.c.	n.c.	n.c.	n.c.	n.c.	n.c.	n.c.	n.c.	n.c.	2
Direct B.1	6.54	2	Part. B	-	-	-	-	0.95	-	0.05	6.21	-	0.33	-	-	-	3
Direct B.2	6.618	2		-	-	-	-	0.92	-	0.08	6.10	-	0.52	-	-	-	11
Direct B.3	5.52	2		-	-	-	-	0.88	-	0.12	4.86	-	0.66	-	-	-	12
Direct B.4	2.556	2		-	-	-	-	0.89	-	0.11	2.27	-	0.29	-	-	-	5
Direct C.1	23.022	2	Part. C	-	-	-	-	0.94	-	0.06	21.64	-	1.38	-	-	-	3
Direct C.2	9.678	2		-	-	-	-	0.91	-	0.09	8.77	-	0.09	-	-	-	8
Direct C.3	16.95	2		-	-	-	-	0.94	-	0.06	16.00	-	0.95	-	-	-	2
Direct C.4	14.91	2		-	-	-	-	0.94	-	0.06	14.08	-	0.83	-	-	-	0
Direct D.1	1.482	3	Part. D	-	-	-	-	0.91	-	0.09	1.35	-	0.13	-	-	-	0
Direct D.2	4.614	2		-	-	-	-	0.93	-	0.07	4.29	-	0.33	-	-	-	5
Direct D.3	4.842	2		-	-	-	-	0.96	-	0.04	4.66	-	0.18	-	-	-	2
Direct D.4	0.882	2		-	-	-	-	0.70	-	0.30	0.61	-	0.27	-	-	-	6
Secondary A	0.18	1	Part. A	n.c.	n.c.	n.c.	n.c.	n.c.	n.c.	n.c.	n.c.	n.c.	n.c.	n.c.	n.c.	n.c.	0
Secondary B	0.528	3	Part. B	Part. D	8.78	6.48	8.82	0.67	0.21	0.11	0.36	0.11	0.06	25	0.28	0.24	1
Secondary C	2.922	3	Part. C	Part. D	6.77	5.035	6.77	0.87	0.08	0.05	2.54	0.23	0.15	22	0.63	0.45	4
Secondary D	5.226	3	Part. D	-	-	-	-	0.93	-	0.07	4.85	-	0.38	-	-	-	3

## Data Availability

Data are available upon request to the corresponding author.
